# Remote ischaemic pre- and delayed postconditioning – similar degree of cardioprotection but distinct mechanisms

**DOI:** 10.1113/expphysiol.2012.064923

**Published:** 2012-03-16

**Authors:** Marina Basalay, Veronika Barsukevich, Svetlana Mastitskaya, Alexander Mrochek, John Pernow, Per-Ove Sjöquist, Gareth L Ackland, Alexander V Gourine, Andrey Gourine

**Affiliations:** 1 Research Centre CardiologyMinsk, Belarus; 2 Neuroscience, Physiology & PharmacologyLondon, UK; 5 Department of Medicine, University College LondonLondon, UK; 3 Department of Cardiology, Karolinska University HospitalStockholm, Sweden; 4 Bioscience, AstraZenecaMölndal, Sweden

## Abstract

Myocardial ischaemia–reperfusion injury can be significantly reduced by an episode(s) of ischaemia–reperfusion applied prior to or during myocardial ischaemia (MI) to peripheral tissue located at a distance from the heart; this phenomenon is called remote ischaemic conditioning (RIc). Here, we compared the efficacy of RIc in protecting the heart when the RIc stimulus is applied prior to, during and at different time points after MI. A rat model of myocardial ischaemia–reperfusion injury involved 30 min of left coronary artery occlusion followed by 120 min of reperfusion. Remote ischaemic conditioning was induced by 15 min occlusion of femoral arteries and conferred a similar degree of cardioprotection when applied 25 min prior to MI, 10 or 25 min after the onset of MI, or starting 10 min after the onset of reperfusion. These RIc stimuli reduced infarct size by 54, 56, 56 and 48% (all *P* < 0.001), respectively. Remote ischaemic conditioning applied 30 min into the reperfusion period was ineffective. Activation of sensory nerves by application of capsaicin was effective in establishing cardioprotection only when elicited prior to MI. Vagotomy or denervation of the peripheral ischaemic tissue both completely abolished cardioprotection induced by RIc applied prior to MI. Cardioprotection conferred by delayed remote postconditioning was not affected by either vagotomy or peripheral denervation. These results indicate that RIc confers potent cardioprotection even if applied with a significant delay after the onset of myocardial reperfusion. Cardioprotection by remote preconditioning is critically dependent on afferent innervation of the remote organ and intact parasympathetic activity, while delayed remote postconditioning appears to rely on a different signalling pathway(s).

Ischaemic heart disease is a major cause of morbidity and mortality in the western world (Lloyd-Jones *et al.* 2009). Reperfusion therapy, which ensures the rapid return of blood flow to the ischaemic myocardium, has been a key advance in the treatment of acute myocardial infarction (Braunwald & Kloner, 1985). However, restoration of blood flow also results in a cascade of harmful events leading to myocardial reperfusion injury (Yellon & Hausenloy, 2007; Prasad *et al.* 2009; Turer & Hill, 2010). Over the past two decades, the pathological mechanisms underlying reperfusion injury have been under intense scrutiny. The currently prevailing consensus states that lethal reperfusion injury – defined as the death of cardiomyocytes still viable at the end of the ischaemic period – is triggered within the first minute(s) of reperfusion (Piper & García-Dorado, 1999; Piper *et al.* 2004; Hausenloy *et al.* 2005; Ovize *et al.* 2010). Zhao *et al.* (2003) demonstrated significant reduction in infarct size in dogs when brief cycles of ischaemia–reperfusion were applied immediately at the onset of reperfusion following a prolonged ischaemic episode (ischaemic postconditioning; IPost). Likewise, in a rabbit model, IPost was effective in reducing infarct size only when initiated at the onset of reperfusion, but conferred no protection when applied 10 min into the reperfusion period (Yang *et al.* 2004). Kin *et al.* (2004), using a rat model of myocardial ischaemia–reperfusion, confirmed that IPost is only effective in protecting myocardium when applied not later than 1 min after the onset of reperfusion.

These results from *in vivo* experiments and numerous data obtained in studies conducted in isolated cardiomyocytes and whole heart preparations using a variety of techniques have led to a general consensus that any treatments of myocardial reperfusion injury can only be effective if applied either prior to or at the immediate onset of reperfusion (reviewed by Gomez *et al.* 2009; Ovize *et al.* 2010). However, Roubille *et al.* (2011) have recently demonstrated in a mouse model that IPost confers significant cardioprotection when applied as late as 30 min after reperfusion onset. These data directly challenged the prevailing concept that lethal myocardial injury occurs in the first minutes of reperfusion. The authors proposed the existence of a ‘dynamic wave-front of reperfusion-induced cell death’, rejecting the idea of an instantaneous reperfusion injury, and suggested that progressive myocardial damage develops over time during the reperfusion period.

Episode(s) of ischaemia–reperfusion in myocardial tissue remote from the index ischaemic myocardium, referred to as remote ischaemic conditioning (RIc), protect against ischaemia–reperfusion injury (Przyklenk *et al.* 1993). Ischaemic myocardium can also be protected by brief episodes of ischaemia–reperfusion applied either before or during ischaemia to peripheral tissue (Gho *et al.* 1996; Kerendi *et al.* 2005), and promising results of recent trials in patients with acute myocardial infarction (Bøtker *et al.* 2010) may facilitate the introduction of RIc procedure(s) into clinical practice.

The present study tested the hypothesis that cardioprotection can be induced by a RIc stimulus applied with a significant delay after the onset of the myocardial reperfusion. We used a rat model of myocardial ischaemia–reperfusion and compared the efficacy of RIc in establishing cardioprotection when RIc is applied prior to, during and at different time points after myocardial ischaemia. Considering that both neural (autonomic) and humoral mechanisms appear to be important for remote ischaemic preconditioning (RPrec; Hausenloy & Yellon, 2008; Lim *et al.* 2010), we also evaluated the relative significance of these pathways in mediating remote ischaemic pre- and delayed postconditioning.

## Methods

All the experiments were performed in accordance with the European Commission Directive 86/609/EEC (European Convention for the Protection of Vertebrate Animals used for Experimental and Other Scientific Purposes) and the UK Home Office (Scientific Procedures) Act (1986) with project approval from the respective Institutional Animal Care and Use Committees.

### Animal preparation

Adult male Wistar rats (280–320 g) were anaesthetized with pentobarbital sodium (induction 60 mg kg^−1^i.p.; maintenance 10 mg kg^−1^ h^−1^i.v.). Adequate anaesthesia was ensured by maintaining stable levels of arterial blood pressure and heart rate and monitored by the absence of a withdrawal response to a paw pinch. The right carotid artery and left jugular vein were cannulated for measurement of arterial blood pressure and administration of anaesthetic, respectively. The trachea was cannulated, and the animal was artificially ventilated with room air using a positive pressure ventilator with a tidal volume of ∼8–10 ml kg^−1^ and a ventilator frequency of ∼60 strokes min^−1^. Partial pressures of O_2_ and CO_2_ as well as pH of the arterial blood were measured regularly and, if required, ventilation was adjusted accordingly to maintain these values within the physiological ranges. A standard lead II ECG was recorded throughout the experiment. The body temperature was maintained at 37.0 ± 0.2°C with a servo-controlled heating pad.

### Myocardial ischaemia–reperfusion

The heart was exposed via a left thoracotomy. A 5–0 monofilament polypropylene suture was passed around the left anterior descending coronary (LAD) artery to induce a temporary occlusion. The animal was subjected to 30 min of myocardial ischaemia induced by LAD artery ligation, followed by 120 min of reperfusion. Successful coronary artery occlusion was confirmed by elevation of the ST segment and an immediate fall in blood pressure by 15–30 mmHg.

### Induction of RIc

The protocol described by Shahid *et al.* (2008) was used. Blood supply to the limbs was interrupted for 15 min by placing vessel clamps on both femoral arteries at the proximal level ∼1 cm below the inguinal ligament. The sham-RIc procedure involved dissection of both femoral arteries, but no occlusion was performed.

### Measurements of infarct size

At the end of the reperfusion period, the LAD artery was re-occluded, and 1 ml of 1.5% Evans Blue dye was injected into the jugular vein for assessment of the area at risk. The animal was then given an anaesthetic overdose (pentobarbital 250 mg kg^−1^i.v.), the heart was excised, and the left ventricle (LV) was isolated, frozen and sectioned into five or six transverse slices from the apex to the base. The slices were weighed and photographed. The area at risk was demarcated by the absence of Evans Blue staining. Left ventricular slices were then incubated with 1% 2,3,5-triphenyltetrazolium chloride in Tris buffer (pH 7.4) for 15 min at 37°C, fixed in 4% formalin for 24 h, and photographed again. Viable myocardium is stained red by 2,3,5-triphenyltetrazolium chloride, whereas necrotic myocardium appears pale yellow. The area at risk and the necrotic area were determined by computerized planimetry, normalized to the weight of each slice, with the degree of necrosis (i.e. infarct size) expressed as the percentage of area at risk.

### Experimental protocols

**Figure 1:**
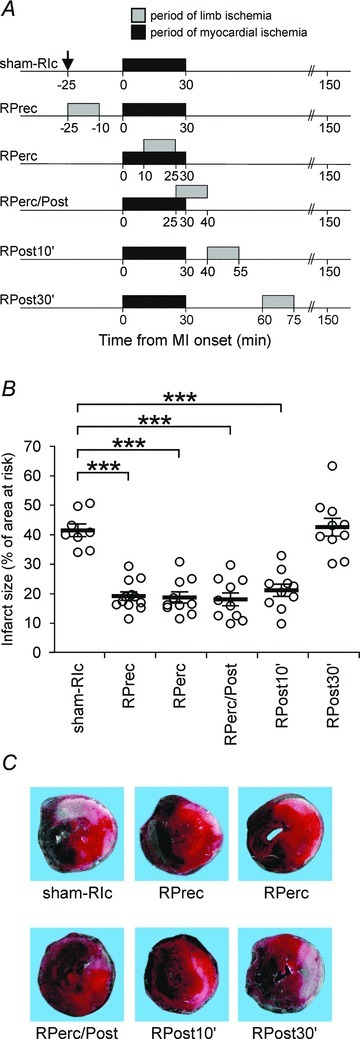
Cardioprotection conferred by remote ischaemic conditioning applied prior to, during and at different time points after myocardial ischaemia (MI) *A*, illustration of the experimental protocols. In all protocols, the model of myocardial ischaemia–reperfusion injury involved 30 min of left coronary artery occlusion followed by 120 min of reperfusion. Remote ischaemic conditioning (RIc) was induced by 15 min occlusion of both femoral arteries. The sham-RIc procedure involved dissection of both femoral arteries without occlusion (arrow). Abbreviations: RPrec, remote preconditioning; RPerc, remote perconditioning; RPerc/Post, remote per-postconditioning; RPost10′, delayed remote postconditioning applied 10 min into reperfusion; and RPost30′, delayed remote postconditioning applied 30 min into reperfusion. *B*, infarct size is presented as a percentage of the area at risk. Remote ischaemic conditioning confers significant cardioprotection when applied 25 min prior to myocardial ischaemia, 10 or 25 min after the onset of myocardial ischaemia or starting 10 min after the onset of reperfusion. Individual data and means ± SEM are shown. ***P < 0.001. *C*, images illustrate representative sections of triphenyltetrazolium chloride-stained hearts from all the experimental groups following 30 min ischaemia and 120 min reperfusion.

**Figure 2:**
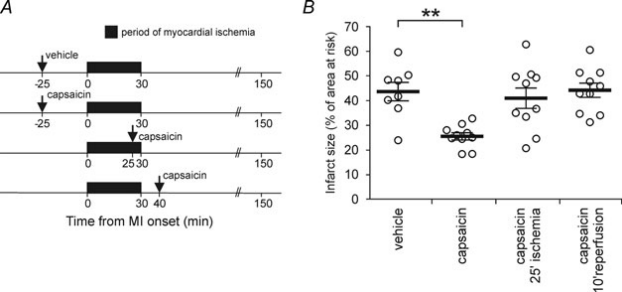
Activation of sensory nerves supplying peripheral tissue by capsaicin is effective in establishing cardioprotection only when applied prior to myocardial ischaemia *A*, illustration of the experimental protocols. Arrow indicates time of subcutaneous administration of capsaicin (3 μg in 10 μl) or vehicle (10% ethanol and 10% Tween 80 in saline; 10 μl) into both hindpaws. *B*, infarct size is presented as a percentage of the area at risk. Capsaicin application confers significant cardioprotection only when applied prior to myocardial ischaemia. Individual data and means ± SEM are shown. **P < 0.01.

**Figure 3:**
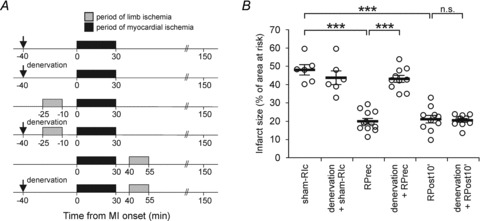
Intact innervation of the remote ischaemic tissue is essential for cardioprotection induced by remote preconditioning but not for delayed remote postconditioning *A*, illustration of the experimental protocols. Arrow indicates time of limb denervation by sectioning sciatic and femoral nerves or sham surgery. *B*, infarct size is presented as a percentage of the area at risk. Denervation of the peripheral ischaemic tissue abolishes cardioprotection induced by remote ischaemic conditioning (RIc) applied prior to myocardial ischaemia, whereas cardioprotection conferred by delayed remote postconditioning (RPost10′) is not affected. Individual data and means ± SEM are shown. ***P < 0.001; n.s., not significant.

**Figure 4:**
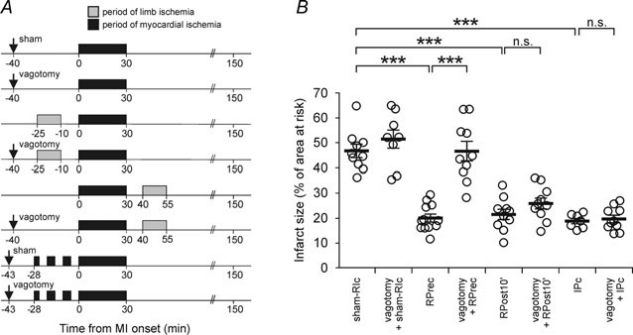
Intact parasympathetic activity is essential for cardioprotection induced by remote preconditioning but not by delayed remote postconditioning or direct myocardial preconditioning *A*, illustration of the experimental protocols. Arrow indicates time of bilateral vagotomy or sham surgery. *B*, infarct size is presented as a percentage of the area at risk. Vagotomy abolishes cardioprotection induced by remote ischaemic conditioning (RIc) applied prior to myocardial ischaemia, whereas cardioprotection conferred by delayed remote postconditioning (RPost10′) or myocardial ischaemic preconditioning (IPc) is not affected. Individual data and means ± SEM are shown. ***P < 0.001; n.s., not significant.

Experimental protocols are illustrated in Figs 1–4.

#### Efficacy of RIc cardioprotection depending upon timing of application of the RIc stimulus (Fig. 1*A*)

Remote ischaemic conditioning was induced by 15 min occlusion of both femoral arteries, followed by reperfusion of the limbs starting at the following time points: (1) 25 min prior to myocardial ischaemia [remote preconditioning (RPrec) group, *n*= 12]; (2) 10 min after the onset of myocardial ischaemia [remote perconditioning (RPrec) group, *n*= 10]; (3) 25 min after the onset of myocardial ischaemia and continuing 10 min into the myocardial reperfusion period [remote per-postconditioning (RPerc/Post) group, *n*= 10]; (4) 10 min after the onset of the reperfusion period [delayed remote postconditioning (RPost10′) group, *n*= 10]; and (5) 30 min after the onset of the reperfusion period [delayed remote postconditioning (RPost30′) group, *n*= 10]. Control animals were subjected to myocardial ischaemia–reperfusion only (*n*= 8).

#### Efficacy of capsaicin-induced cardioprotection depending upon timing of capsaicin application (Fig. 2*A*)

There is evidence that activation of C fibre afferents by topical application of capsaicin to the peripheral tissue prior to myocardial ischaemia–reperfusion limits myocardial injury, mimicking the effect of remote preconditioning (Jones *et al.* 2009). Capsaicin (3 μg in 10 μl) or vehicle (10% ethanol and 10% Tween 80 in saline; 10 μl) were injected subcutaneously into both hindpaws at the following time points: (1) 25 min prior to myocardial ischaemia (*n*= 10); (2) 25 min after the onset of myocardial ischaemia (*n*= 10); or (3) 10 min after the onset of reperfusion (*n*= 10).

#### Efficacy of RIc cardioprotection depending upon intact innervation of the remote ischaemic tissue (Fig. 3*A*)

The femoral nerves were exposed after separation from the femoral artery and vein ∼1 cm below the inguinal ligament. The sciatic nerves were exposed following blunt dissection of the biceps femoris. Femoral arteries were occluded for 15 min commencing 25 min prior to myocardial ischaemia (RPrec group, *n*= 12) or 10 min after the onset of myocardial reperfusion (RPost10′ group, *n*= 10). Femoral and sciatic nerves were sectioned bilaterally 40 min prior to myocardial ischaemia in animals that were treated as follows: (1) subjected to sham-RIc (denervation/sham-RIc group, *n*= 6); (2) subjected to occlusion of the femoral arteries commencing 25 min prior to myocardial ischaemia (denervation/RPrec group, *n*= 10); and (3) subjected to occlusion of the femoral arteries commencing 10 min after the onset of myocardial reperfusion (denervation/RPost10′ group, *n*= 10). In sham-operated animals, dissection of the respective nerves was performed without sectioning (control, *n*= 6).

#### Efficacy of RIc cardioprotection depending upon intact parasympathetic activity (Fig. 4*A*)

Activity of the autonomic nervous system is essential for remote preconditioning, because this phenomenon is abolished under ganglionic blockade following systemic administration of hexamethonium (Loukogeorgakis *et al.* 2005), which blocks transmission in sympathetic and parasympathetic ganglia. Given the protective effect of the vagal tone against myocardial injury (Mioni *et al.* 2005; Katare *et al.* 2009), we assessed whether RIc applied prior to or after myocardial ischaemia requires an intact parasympathetic supply to the heart. Both vagi were exposed at the level of the neck via the ventral approach and sectioned bilaterally 40 min prior to myocardial ischaemia in animals that were treated as follows: (1) subjected to sham-RIc (vagotomy/sham-RIc group, *n*= 8); (2) subjected to occlusion of the femoral arteries commencing 25 min prior to myocardial ischaemia (vagotomy/RPrec group, *n*= 10); and (3) subjected to occlusion of the femoral arteries commencing 10 min after the onset of reperfusion (vagotomy/RPost10′ group, *n*= 10). In sham-operated animals, dissection of vagi was performed without sectioning (control, *n*= 10).

#### Efficacy of myocardial ischaemic preconditioning (IPc) depending upon intact parasympathetic activity (Fig. 4*A*)

Vagi were sectioned bilaterally 15 min prior to the IPc procedure. Ischaemic preconditioning was elicited by three brief periods of ischaemia (first 3 min, followed by two episodes lasting 5 min each) separated by 5 min periods of reperfusion before myocardial ischaemia (*n*= 7). In sham-operated animals, dissection of vagi was performed without sectioning (control, *n*= 10).

### Statistical analysis

Data are reported as means ± SEM. Data were compared by ANOVA followed by the Tukey–Kramer *post hoc* test to determine the main group effect. Values of *P* < 0.05 were considered to be significant.

## Results

There were no differences in the areas at risk between groups of animals recruited into any of the protocols. Figures 1–4 illustrate infarct size data displayed as percentages of the area at risk.

### Efficacy of RIc cardioprotection depending upon timing of application of the RIc stimulus

In this protocol, the mean infarct size of the control group was 41 ± 2% (Fig. 1*B* and *C*). Remote ischaemic preconditioning induced by 15 min occlusion of both femoral arteries, followed by 10 min of reperfusion applied prior to myocardial ischaemia conferred significant cardioprotection, as evident from a marked reduction in infarct size (19 ± 1%, *P* < 0.001; Fig. 1*B* and *C*). A similar degree of cardioprotection was achieved when a 15 min period of limb ischaemia was applied either 10 or 25 min after the onset of myocardial ischaemia and continuing 10 min into reperfusion or starting 10 min after the onset of the reperfusion period (all differences are significant with *P* < 0.001; Fig. 1*B*). Remote ischaemic conditioning applied 30 min after the onset of reperfusion had no effect on infarct size. There were no differences in mean arterial pressure and heart rate between groups of animals during ischaemia and reperfusion (Table S1). Thus, the peripheral RIc stimulus is equally efficient in protecting the heart when applied before myocardial ischaemia, during ischaemia and as late as 10 min after restoration of the blood flow through the compromised myocardium.

### Efficacy of capsaicin-induced cardioprotection depending upon timing of capsaicin application

Administration of capsaicin into the hindpaws 25 min prior to myocardial ischaemia markedly reduced the infarct size (by 43%, *P* < 0.01; Fig. 2*B*). Injection of capsaicin 25 min after an onset of myocardial ischaemia or 10 min into the reperfusion period had no effect on infarct size (Fig. 2*B*). There were no differences in systemic haemodynamic variables between groups of animals during ischaemia and reperfusion (Table S1). These data suggest that activation of sensory nerves and recruitment of the neural pathway(s) is effective in establishing cardioprotection only when applied prior to myocardial ischaemia.

### Efficacy of RIc cardioprotection depending upon intact innervation of the remote ischaemic tissue

Complete denervation of the limbs by sectioning the sciatic and femoral nerves was found to abolish cardioprotection induced by RPrec (43 ± 1 *versus* control 44 ± 3%; Fig. 3*B*). Interestingly, denervation of the limbs had no effect on cardioprotection conferred by the RIc stimulus applied 10 min after the onset of reperfusion (Fig. 3*B*). These data are in agreement with the results of the capsaicin experiment and imply that the neural pathway of cardioprotection, which involves sensory innervation of remote tissue, is recruited if the RIc stimulus is applied prior to myocardial ischaemia.

### Efficacy of RIc and myocardial IPc cardioprotection depending on intact parasympathetic activity

Bilateral vagotomy completely abolished cardioprotection induced by RIc applied 25 min prior to myocardial ischaemia (Fig. 4*B*). Vagotomy had no effect on cardioprotection conferred by RIc applied 10 min after the onset of reperfusion or cardioprotection established by myocardial IPc (Fig. 4*B*). In rats anaesthetized with pentobarbital, chronotropic vagal tone is significantly reduced (O’Leary & Jones, 2003); therefore, vagotomy had no significant effect on heart rate throughout the experimental protocol (Table S1). These observations indicate that the neural pathway of cardioprotection, which involves parasympathetic innervation of the heart, operates only when activated prior to myocardial ischaemia.

## Discussion

The results of the present study demonstrate, for the first time, that a similar degree of cardioprotection could be achieved by ischaemia–reperfusion of the remote tissue applied prior to, during or as late as 10 min after the onset of myocardial reperfusion. Pathways that confer cardioprotection in response to pre- and delayed postconditioning appear to be distinct. Remote preconditioning cardioprotection is critically dependent on afferent innervation of the remote organ and intact parasympathetic activity, while delayed remote postconditioning appears to rely on a different mechanism(s).

### Time-dependent postischaemic cardioprotection

Here we show that the time window for cardioprotection conferred by ischaemia–reperfusion of the remote tissue is significantly wider than was previously thought. By extension, a proportion of cardiomyocytes in the area at risk remains viable well into the reperfusion period and can be rescued by application of an appropriate therapeutic procedure, such as delayed RPost, as shown in the present study. Moreover, the degree of cardioprotection established by the RIc stimulus applied prior to, during and as late as 10 min after the onset of myocardial reperfusion was found to be remarkably similar. These results are supported by recent observations of Roubille *et al.* (2011) and indicate that the death of cardiomyocytes is not triggered shortly after restoration of the blood flow (Griffiths & Halestrap, 1995; Griffiths *et al.* 1998; Piper & García-Dorado, 1999; Di Lisa *et al.* 2001; Hausenloy *et al.* 2004, 2005; Piper *et al.* 2004; Argaud *et al.* 2005; Ovize *et al.* 2010) but is an ongoing process, which also occurs during the later stages of reperfusion.

The data obtained in the present study are largely in agreement with the results reported by Roubille *et al.* (2011), who observed a significant reduction in infarct size by delayed myocardial IPost applied 30 min after the onset of reperfusion. The key finding was the similar degree of cardioprotection conferred by delayed myocardial IPost (Roubille *et al.* 2011) and delayed RPost (present study). The difference in the time window for delayed cardioprotection between the two studies may be related to the fact that different animal species (rats *versus* mice) were used. Also, as we found no evidence that a neural pathway confers cardioprotection elicited by delayed RPost, a humoral mechanism appears to mediate this phenomenon (discussed in the following subsection). As RPost10′ in the present study involved occlusion of both femoral arteries for 15 min, only a minimal amount of humoral factor(s), if any, would be expected to be released from the ischaemic peripheral tissue into the systemic circulation for up to 25 min after restoration of the blood flow through the compromised myocardium. Thus, the time windows for direct and remote postconditioning cardioprotection may well be very similar.

### Neural and humoral mechanisms of cardioprotection

Data obtained in the present study also contribute in a significant manner to an ongoing debate regarding the role of humoral *versus* neural mechanisms in establishing cardioprotection conferred by a remote preconditioning stimulus. The mechanism of RPrec-induced cardioprotection was suggested to involve humoral factor(s) produced during ischaemia–reperfusion of the remote tissue and released into the systemic circulation (Konstantinov *et al.* 2005; Hausenloy & Yellon, 2008; Shimizu *et al.* 2009; Kingma *et al.* 2011) or a neural component (Gho *et al.* 1996; Dong *et al.* 2004; Loukogeorgakis *et al.* 2005; Hausenloy & Yellon, 2008; Jones *et al.* 2009; Steensrud *et al.* 2010), or both (Lim *et al.* 2010).

Our results obtained in the experiments involving denervation of the remote organ, capsaicin application and bilateral vagotomy suggest that both neural and humoral mechanisms may be equally potent in establishing RIc cardioprotection. Their relative significance appears to be critically dependent upon the timing of application of the RIc stimulus. Indeed, in accord with the existing evidence (Dong *et al.* 2004; Lim *et al.* 2010) denervation of the remote organ abolished cardioprotection elicited by RIc applied prior to myocardial ischaemia. However, such denervation had no effect on cardioprotection conferred by RIc applied during myocardial reperfusion. Likewise, RPrec failed to establish cardioprotection following bilateral vagotomy, whereas the cardioprotective effects of delayed RPost or myocardial IPc were not affected. Finally, activation of limb C fibre afferents by topical application of capsaicin (Jones *et al.* 2009) was found to reduce myocardial injury dramatically, but only when capsaicin was applied before myocardial ischaemia. These observations suggest that the neural pathway of cardioprotection, which involves afferent innervation of the remote tissue and parasympathetic activity, is effective only when activated prior to myocardial ischaemia. Cardioprotection established by the RIc stimulus applied during the reperfusion period appears to recruit a completely different mechanism, which is likely to be humoral in nature. Identification of the humoral factor(s) is currently being tackled by many research groups and lies beyond the scope of the present investigation. However, our results may help to reconcile the data reported in the above-mentioned studies, because both neural and humoral pathways of cardioprotection can be recruited, depending on certain conditions (e.g. timing of RIc stimulus application).

### Lethal reperfusion injury

There is a general consensus that lethal reperfusion injury is triggered within the first few minutes following reopening of the occluded coronary artery (Piper & García-Dorado, 1999; Piper *et al.* 2004; Hausenloy *et al.* 2005; Ovize *et al.* 2010). In addition to the evidence mentioned in the Introduction, this idea is supported by experimental studies that implicate opening of the mitochondrial permeability transition pore (mPTP) within the first minutes (∼5 min) of reperfusion (Griffiths & Halestrap, 1995; Griffiths *et al.* 1998; Di Lisa *et al.* 2001) as the key event responsible for cardiomyocyte death (Crompton *et al.* 1987; Borutaite *et al.* 2003; Green & Kroemer, 2004; Baines, 2009). Evidence supporting a key role of mPTP opening in lethal reperfusion injury was also obtained in transgenic animals (Lim *et al.* 2007). Signalling pathways and mechanisms implicated in cardioprotection during myocardial ischaemia–reperfusion, including K_ATP_ channels, reactive oxygen species, nitric oxide and others, were suggested to be active upstream of mPTP. Inhibition of mPTP is also thought to be the main mechanism of the beneficial action of prosurvival kinases, such as reperfusion injury salvage (RISK) kinase (Juhaszova *et al.* 2004; Hausenloy *et al.* 2005) and survivor activating factor enhancement (SAFE) kinase (Boengler *et al.* 2010). The data obtained in the present study suggest that reperfusion cell death is not entirely mediated via mPTP opening or that not all cardiomyocytes in the area at risk display the same dynamics of mPTP opening or that cardiomyocytes remain viable and can be rescued after (partial) mPTP opening.

### Clinical relevance

Identification of possible targets and development of therapeutic strategies to protect the heart following acute myocardial infarction has been a challenging task. Demonstration that IPc of the heart confers significant cardioprotection against lethal ischaemia–reperfusion injury by recruitment of innate protective mechanisms (Murry *et al.* 1986) triggered significant interest in identification of these mechanisms. Subsequently, Zhao *et al.* (2003) described a similar cardioprotective effect of IPost, elicited by cycles of coronary artery occlusion–reperfusion applied at the immediate onset of reperfusion.

However, widespread routine clinical application of myocardial IPc and IPost in patients with acute myocardial infarction is limited by the unpredictable onset of the ischaemic event or by technical difficulties requiring complex invasive procedures to apply direct myocardial IPost. Remote ischaemic conditioning represents an effective and safe strategy for limiting myocardial injury (Bøtker *et al.* 2010) and may be easily applied via a limb cuff to a majority of patients in different clinical settings. The timing of treatment(s) is an important issue, and results of the present study demonstrate that the RIc stimulus is highly effective in reducing myocardial injury even if applied with a significant delay after restoration of blood flow through the compromised myocardium.

### Conclusion

Remote ischaemic conditioning effectively protects the heart against ischaemia–reperfusion injury even if applied with a significant delay after the onset of myocardial reperfusion. Remote preconditioning cardioprotection is critically dependent on afferent innervation of the remote organ and intact parasympathetic activity, while delayed remote postconditioning recruits a different signalling pathway(s). These results support data recently reported by Roubille *et al*. (2011) and together challenge the current theory that lethal reperfusion injury is triggered within the first minutes of reperfusion.

## Supporting Information

The following supporting information is available in the online version of this article:
