# Supplementary material for: Remote ischaemic pre- and delayed postconditioning – similar degree of cardioprotection but distinct mechanisms
Source: Exp Physiol. 2012 Mar 16;97(8):908–17. doi: 10.1113/expphysiol.2012.064923 (PMC3470925; doi:10.1113/expphysiol.2012.064923)
Supplement: Supplementary file 1 [file eph0097-0908-SD1.pdf]

Supplementary Table

	Variable	Basal	Pre-ischaemia	End of ischaemia	Reperfusion (min)			
					30	60	90	120
**Experiment 1**								
Sham-RIc (n=8)	MAP	108±6	106±6	105±7	95±7	89±6	87±5	87±5
	HR	422±16	412±11	421±12	436±15	445±10	446±9	442±8
RPrec (n=12)	MAP	102±5	102±7	99±6	96±5	90±5	87±6	82±4
	HR	418±8	421±11	432±12	428±12	428±12	428±13	424±9
RPerc (n=10)	MAP	104±5	99±5	97±3	92±2	88±6	90±5	86±4
	HR	429±10	429±10	438±12	425±14	444±12	436±15	440±14
RPerc/Post (n=10)	MAP	99±7	103±7	98±6	97±5	95±5	88±5	91±5
	HR	410±16	412±16	411±14	398±16	395±16	411±14	419±13
RPost10' (n=10)	MAP	101±2	106±2	91±11	87±6	89±5	82±4	89±3
	HR	412±14	402±14	421±7	413±16	422±15	418±16	434±11
RPost30' (n=10)	MAP	104±18	101±18	95±6	88±4	86±4	84±5	84±4
	HR	409±12	409±14	419±11	415±13	422±13	412±17	417±14
**Experiment 2**								
Vehicle (n=8)	MAP	99±8	96±8	93±7	90±6	87±6	84±5	87±4
	HR	405±17	405±17	421±15	428±13	431±9	435±11	422±5
Capsaicin (n=10)	MAP	104±3	105±2	92±4	88±3	90±7	88±6	86±4
	HR	402±14	413±12	407±13	405±15	422±18	427±18	425±17
Capsaicin	MAP	99±5	95±5	98±4	82±3	82±3	84±3	76±3
25'ischaemia (n=10)	HR	407±14	405±14	421±13	409±12	432±12	426±11	433±11
Capsaicin	MAP	101±4	99±4	95±3	79±4	79±3	81±3	82±3
10'reperfusion (n=10)	HR	417±9	413±9	436±9	414±13	424±11	429±12	435±11

Supplementary Table (contd)

**Experiment 3**								
Sham-RIc (n=6)	MAP	101±9	99±9	95±10	85±4	84±3	82±5	87±5
	HR	413±20	413±20	418±17	428±12	426±12	430±14	443±7
Denervation+	MAP	96±3	96±5	92±4	86±1	80±4	75±3	80±7
Sham- RIc (n=6)	HR	415±8	415±9	442±13	420±14	425±19	430±23	452±11
RPrec (n=12)	MAP	102±5	102±7	99±6	96±5	90±5	87±6	82±4
	HR	418±8	421±11	432±12	428±12	428±12	428±13	424±9
Denervation+RPrec	MAP	106±4	99±4	92±5	85±4	87±3	88±3	86±4
(n=10)	HR	417±13	404±19	436±11	427±18	427±17	440±18	441±16
RPost10' (n=10)	MAP	101±2	106±2	91±11	87±6	89±5	82±4	89±3
	HR	412±14	402±14	421±7	413±16	422±15	418±16	434±11
Denervation+	MAP	109±3	99±2	92±4	88±3	85±3	88±3	81±4
RPost10' (n=10)	HR	411±28	408±14	419±12	413±13	419±11	422±10	432±9
**Experiment 4**								
Sham-RIc (n=10)	MAP	102±5	97±5	94±5	92±5	87±6	92±4	88±4
	HR	422±17	406±17	413±17	426±23	424±22	437±16	439±15
Vagotomy +Sham-	MAP	88±6	97±4	89±3	102±7	104±7	98±6	94±6
RIc (n=8)	HR	432±21	434±11	451±11	457±13	451±14	450±8	451±9
RPrec (n=12)	MAP	102±5	102±7	99±6	96±5	90±5	87±6	82±4
	HR	418±8	421±11	432±12	428±12	428±12	428±13	424±9
Vagotomy+RPrec	MAP	99±3	115±7	114±8	103±7	96±6	98±4	96±4
(n=10)	HR	431±12	465±4	472±5	453±9	456±5	459±3	458±3
RPost10' (n=10)	MAP	101±2	106±2	91±11	87±6	89±5	82±4	89±3
	HR	412±14	402±14	421±7	413±16	422±15	418±16	434±11
Vagotomy+RPost10'	MAP	104±5	101±5	95±6	104±8	98±7	93±5	90±5
(n=10)	HR	413±14	426±14	429±15	419±16	418±15	423±15	420±15
Sham-IPc (n=7)	MAP	102±6	99±6	97±7	92±3	95±3	85±4	81±5
	HR	430±14	444±6	432±6	445±8	441±7	445±7	444±8
Vagotomy+IPc (n=10)	MAP	100±5	104±8	101±6	96±6	91±4	87±5	88±6
	HR	433±12	460±10	461±10	464±9	464±10	463±11	459±10

Abbreviations: RIc – remote ischaemic conditioning; RPrec – remote ischaemic preconditioning; RPerc – remote ischaemic perconditioning; RPost – remote ischaemic postconditioning; IPc – myocardial ischaemic preconditioning; HR – heart rate (beats/min); MAP – mean arterial blood pressure (mmHg).
